# Deregulation of the IL-1β axis in chronic recurrent multifocal osteomyelitis

**DOI:** 10.1186/1546-0096-12-30

**Published:** 2014-07-17

**Authors:** Roberta Scianaro, Antonella Insalaco, Luisa Bracci Laudiero, Rita De Vito, Marco Pezzullo, Anna Teti, Fabrizio De Benedetti, Giusi Prencipe

**Affiliations:** 1 Rheumatology Unit, Bambino Gesù Children’s Hospital, Rome, Italy; 2 Division of Pathology, Bambino Gesù Children’s Hospital, Rome, Italy; 3 Core Facilities, Bambino Gesù Children’s Hospital, Rome, Italy; 4 Department of Biotechnological and Applied Clinical Sciences, University of L’Aquila, L’Aquila, Italy

**Keywords:** Chronic recurrent multifocal osteomyelitis, Autoinflammatory disorders, Inflammasome, Interleukin-1β, Osteoclasts

## Abstract

**Background:**

This study aims to investigate the inflammasome response in peripheral blood mononuclear cells (PBMCs) and the expression of inflammasome components in bone biopsies from patients with chronic recurrent multifocal osteomyelitis (CRMO).

**Methods:**

The expression of inflammasome components mRNAs was evaluated in PBMCs isolated from 15 CRMO patients and 13 healthy controls by quantitative real-time PCR. The Interleukin (IL)-1β released in the medium of PBMC cultures after treatment with lipopolysaccharides (LPS) alone or LPS and ATP was measured by ELISA. Immunohistochemical staining for Apoptosis-associated Speck-like protein (ASC), caspase-1 (CASP-1), Nod-like receptor protein-3 (NLRP3) and IL-1β expression was performed in bone biopsies from CRMO patients.

**Results:**

mRNA levels of *ASC*, *CASP-1* and *IL-1β* were significantly higher in freshly isolated PBMCs from CRMO patients in active disease than in healthy controls. *CASP-1* and *IL-1β* transcript levels were significantly higher also in PBMCs from CRMO patients in remission compared to healthy controls. PBMCs from CRMO patients in active disease stimulated in vitro with LPS showed a significant increase in IL-1β release compared to healthy control cells. Immunohistochemistry staining of bone tissue revealed the expression of inflammasome components in CRMO osteoclasts.

**Conclusions:**

Our data suggest that an abnormal regulation of IL-1β axis may be involved in CRMO pathogenesis.

## Background

Chronic recurrent multifocal osteomyelitis (CRMO) is a rare skeletal disorder, predominantly affecting children, that presents with bone pain, fever and sterile multifocal osteolytic bone lesions, characterized by inflammatory infiltrate and activated osteoclasts [1].

Osteoclasts belong to the monocyte-macrophage lineage, are responsible for bone resorption and are regulated by pro-inflammatory cytokines [2]. CRMO is usually sporadic, but there is evidence of a genetic component, as several reports described the disease in siblings and in monozygotic twins [3]. Moreover, a monogenic disease associated with CRMO, termed Majeed’s Syndrome, is known [4].

Although CRMO is considered an autoinflammatory disease, the pathogenetic mechanisms are still unknown. Recent findings demonstrate, in a murine model of CRMO, an inflammasome-independent role for the interleukin-1 (IL-1) pathway in the disease pathogenesis [5]. In patients with CRMO, an imbalance between pro-inflammatory (IL-6, TNF-α) and anti-inflammatory cytokines (IL-10) has been hypothesized to be involved [6]. Autoinflammatory diseases are characterized by deregulation of the innate immune system, often involving the IL-1β pathway [7]. Release of IL-1β is induced by inflammasomes, multi-protein cytoplasmic complexes, composed by pattern recognition receptors, including Toll-like receptors (TLRs) and Nod-like receptors, the adaptor protein Apoptosis-associated speck-like protein containing a CARD (ASC) and pro-caspase-1, that sense microbial molecules and endogenous danger signals [8].

IL-1β secretion requires two signals. The first signal is provided by microbial or endogenous molecules that activate NF-κB and induce, through a TLR, expression and synthesis of the inactive IL-1β precursor (pro-IL-1β). The second signal is provided by adenosine triphosphate (ATP), certain bacterial toxins or particulate matter and directly activates inflammasomes, resulting in pro-IL-1β cleavage and IL-1β secretion [8].

The aim of this study was to examine the inflammasome response in peripheral blood mononuclear cells (PBMCs) and the expression of inflammasome components in bone biopsies from CRMO patients.

## Methods

### Subjects

Peripheral blood was obtained from 15 patients with CRMO. The diagnosis of CRMO was based on clinical presentation, laboratory and imaging data and bone biopsy. Bone biopsies (obtained from all patients, except one) showed evidence of sterile bone inflammation.

At the time of the study, 7 patients had active disease, defined by patients’ reported bone and/or joint pain, increased C-reactive protein (CRP) (normal <0.5 mg/dl) or erythrocyte sedimentation rate (ESR) (normal <20 mm/h) and lesions demonstrating increased uptake of technetium-99 m in bone scan. The main characteristics of CRMO patients in active disease were: 5 females and 2 males, mean age at onset 9.57 ± 5.16 years, mean age at sampling 10.31 ± 5.72 years, number of bone lesions from 1 to 6 (median 3); 5 patients were treated with non steroidal anti-inflammatory drugs (NSAID) and 2 were not receiving any treatment. The remaining 8 patients who did not meet the criteria for active disease were defined as having remission: 4 females and 4 males, mean age at onset 7.22 ± 4.64 years, mean age at sampling 10.36 ± 5.38 years; 2 patients were treated with methotrexate, 1 with sulfasalazine and NSAID and the remaining 5 were not receiving any treatment.

Thirteen healthy children comparable for age, hospitalized for minor surgical procedures, were used as controls. Blood samples were collected before they underwent surgery. They did not have evidence of infection or inflammation or increased CRP at time of blood sampling. In addition, peripheral blood was obtained also from 7 children with active juvenile idiopathic arthritis (JIA) (mean age at sampling 8.14 ± 2.54 years). Blood samples were taken after informed consent of the parents.

The study was approved by the Institutional Ethical Committee.

### PBMC isolation and in vitro stimulation

PBMCs were separated from blood by Ficoll/Histopaque (Sigma-Aldrich). Freshly isolated PBMCs were incubated in DMEM (Gibco) plus 10% FBS with 10 ng/ml lipopolysaccharides (LPS, E. coli serotype 055:B5, Sigma-Aldrich) for 3 hours, or stimulated with 10 ng/ml LPS for 2 hours, followed by stimulation with 2 mM ATP (Sigma-Aldrich) for 1 hour.

### Cytokine detection

Levels of IL-1β in PBMC supernatants were quantified by ELISA (R&D System). Circulating IL-1β and IL-6 levels were measured in plasma collected from patients and controls by ELISA, using commercial kits (R&D System) according to the manufacturer’s instructions.

### RNA isolation and real-time PCR

Total RNA was extracted using the Qiagen-RNeasy Mini kit (Qiagen) from 10^6^ PBMCs and cDNA was obtained using the Superscript Vilo kit (Invitrogen). Real-time PCR assays were performed using TaqMan Universal PCR Master mix (Applied Biosystems) and the following gene-expression assays: human *IL-1β, TNF-α, CASP-1, ASC and NLRP3* (Applied Biosystem). Gene expression data were normalized using *HPRT*, as endogenous control. Data are expressed as arbitrary units (AU) using the 2^-ΔCt^ transformation method.

### Immunohistochemistry

Bone-biopsy specimens were available from three CRMO patients and were evaluated by immunohistochemistry. A bone-biopsy from a patient with acute lymphoblastic leukemia in remission was used as tissue control. Formalin-fixed, decalcified and embedded in paraffin bone-biopsy specimens were immunostained with antibodies to human ASC (LifeSpan BioSciences), NLRP3 (Sigma-Aldrich), CASP-1 (Cell Signaling), IL-1β (Cell Signaling) and IL-6 (Abcam), revealed by LSAB2 System-HRP (DakoCytomation) and counterstained with hematoxylin.

### Statistical analysis

Quantitative data were expressed as mean ± SD or as median and interquartile range (IQR). Statistical analysis was performed by the Mann–Whitney *U* test. *P* values less than 0.05 were considered significant.

## Results

**Figure 1:**
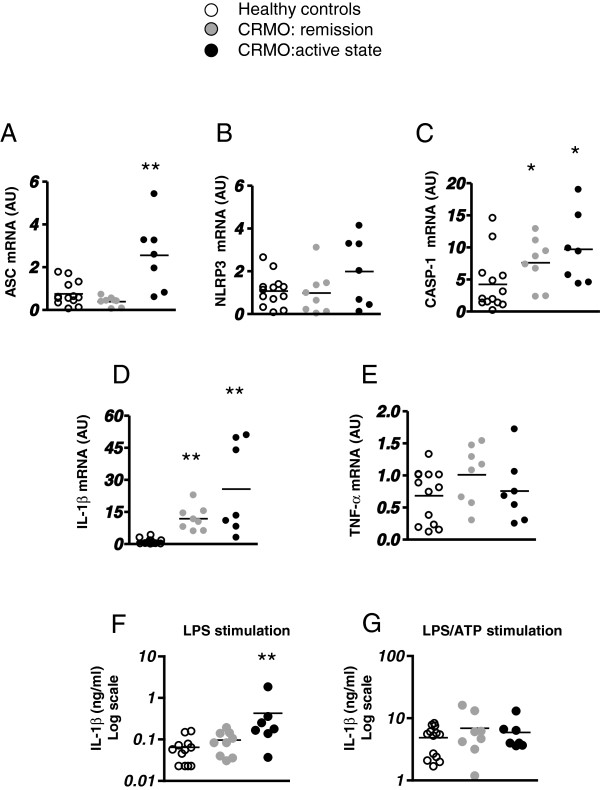
**Characterization of PBMCs from CRMO patients. (A-E)** Real Time-PCR analysis of gene products involved in the regulation of IL-1β, including *ASC*, *NLRP3*, *CASP-1*, *IL-1β* and of the pro-inflammatory cytokine *TNF-α* in freshly isolated PBMCs obtained from CRMO patients in remission (gray dots) or with active disease (black dots) and healthy controls (white dots). Values are presented as arbitrary unit (AU). **(F-G)** IL-1β released in supernatants by PBMCs isolated from CRMO patients in remission or in active disease and from healthy controls, after stimulation with 10 ng/ml LPS for 3 hours **(F)** or 10 ng/ml LPS for 2 hours followed by treatment with 2 mM ATP for 1 hour **(G)**. IL-1β was measured by ELISA. Black lines represent the mean value. *p < 0.05, **p < 0.01 vs healthy controls.

*ASC*, *CASP-1* and *IL-1β* mRNA levels were significantly higher in PBMCs freshly isolated from CRMO patients during active disease compared to PBMCs from healthy controls. The mRNA expression of *CASP-1* and *IL-1β* was also significantly higher in PBMCs from patients in remission compared to healthy controls (Figure 1A-D).

No significant difference in *TNF-α* expression was observed between the two patient groups and healthy controls (Figure 1E).

When we analyzed cytokine levels in plasma, we failed to measure detectable levels of circulating IL-1β (data not shown). We found that, compared to healthy controls [median value 0.156 pg/ml, Interquartile range (IQR) 0.156-0.612], circulating levels of IL-6 were similar in patients in remission (median value 0.327 pg/ml, IQR 0.156-1.825) (p = 0.14), while they were significantly increased in patients during active disease (median value 8.48 pg/ml, IQR 4.93-23.72) (p = 0.0004).

To evaluate inflammasome activation in PBMCs from CRMO patients, freshly isolated PBMCs were stimulated in vitro with LPS alone or LPS plus ATP and the IL-1β released in the medium was measured. After LPS stimulation, the amount of IL-1β released by PBMCs from CRMO patients with active disease was significantly higher when compared to patients during remission or to healthy controls (Figure 1F). In contrast, cells from patients with active JIA stimulated with LPS alone produced amount of IL-1β comparable to those released by healthy controls (0.067 ng/ml ±0.061 versus 0.064 ng/ml ±0.046, p > 0.5). Stimulation with LPS plus ATP led, as expected, to a marked increase in IL-1β release, with no significant differences between the three groups (Figure 1G). In patients with active disease, *IL-1β* mRNA levels in PBMCs freshly isolated or IL-1β release following in vitro LPS stimulation were not significantly associated with the number of bone lesions (R = 0.480, p = 0.27; R = 0.566, p = 0.17, respectively) or ESR (R = 0.141, p = 0.75; R = 0.50, p = 0.24, respectively). In two patients, we analyzed PBMC *IL-1β* mRNA levels before and after (16 and 18 days, respectively) pamidronate administration: a 2-fold reduction in *IL-1β* mRNA levels was observed (pre-treatment 49.7 and 50.9; post-treatment 28.1 and 23.3 arbitrary unit, respectively).

**Figure 2:**
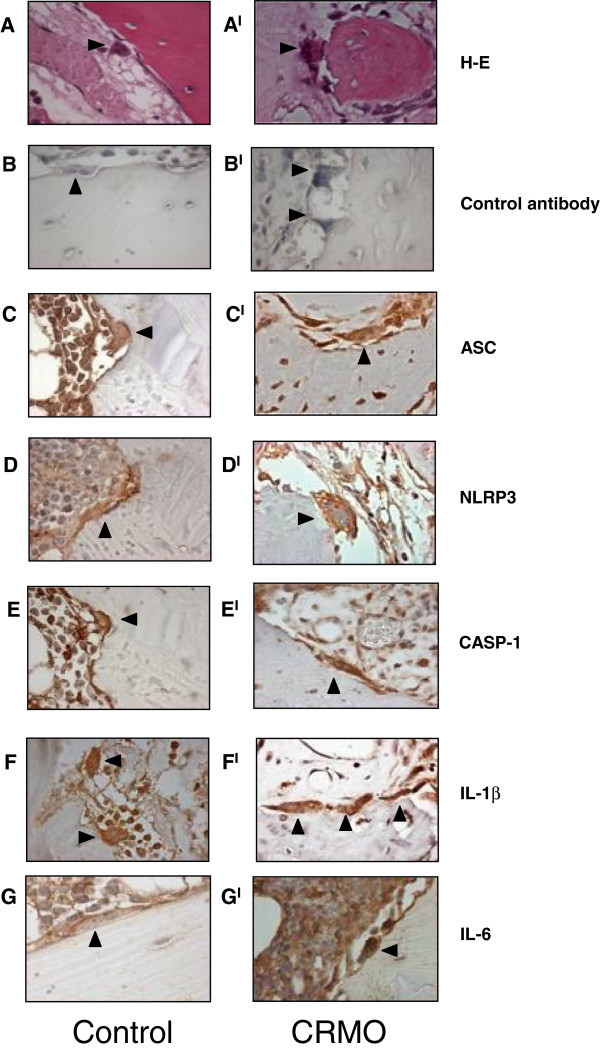
**Expression of inflammasome components and IL-6 in bone biopsies from CRMO patients and from one tissue control.** Representative immunohistochemical staining of decalcified human bone biopsy specimens from a tissue control **(A-B-C-D-E-F-G)** and patients with CRMO **(A**^**I**^**-B**^**I**^**-C**^**I**^**-D**^**I**^**-E**^**I**^**-F**^**I**^**-G**^**I**^**)**. Bone sections were stained with hematoxylin-eosin, a secondary antibody only or with primary antibodies as indicated. Magnification: X63. Arrows: osteoclasts.

The presence of activated osteoclasts is a typical feature of bone lesions in CRMO. Because of their potential pathogenic role in CRMO, we performed the immunohistochemical staining of bone biopsy specimens from CRMO patients (n = 3), (Figure 2A^I^-F^I^) and from one tissue control (Figure 2 A-F) with antibodies to ASC, NLRP3, CASP-1 and IL-1β. In bone tissue from CRMO patients and one control, the expression of the three inflammasome components as well as of IL-1β was detected, demonstrating that also osteoclasts expressed components of the inflammasome machinery.

## Discussion

We demonstrated an abnormal regulation of the IL-1β axis and its secretory machinery in CRMO patients. PBMCs from CRMO patients obtained during the active disease expressed higher mRNA levels of inflammasome key components, *ASC* and *CASP-1*, and higher levels of *IL-1β* mRNA. Moreover, PBMCs from CRMO patients, cultured in vitro, showed a higher IL-1β release after treatment with LPS alone. These results are consistent with a deregulation of the IL-1β processing machinery. Indeed, in patients with cryopyrinopathies, caused by gain of function NLPR3-mutations, increased IL-1β release following stimulation with LPS alone is a typical feature of the disease [9]. IL-1β release can result in autocrine stimulation and in the secondary induction of other cytokines, such as IL-6. We indeed found that high circulating IL-6 levels are present during active disease. *In vivo*, neutralization of excessive IL-1β in cryopyrinopathies and in deficiency of interleukin-1 receptor antagonist (DIRA) results in decrease in IL-6 production [10-12].

We could not detect circulating IL-1β. However, detection of circulating IL-1β has proven to be extremely difficult even in diseases in which undoubtedly excessive IL-1β production is known to be involved, including cryopirinopathies and systemic JIA [13,14]. Therefore, the absence of detectable levels of IL-1β does not rule out a pathogenic role for this cytokine. Indeed, satisfactory responses to anakinra have been reported in one patient with synovitis-acne-pustulosis-hyperostosis-osteitis syndrome (SAPHO) [15], and in two patients with Majeed syndrome [16]. One transient response has been reported in one patient with CRMO [17]. These observations, together with our results, point to a role for IL-1β in the pathogenesis of CRMO and further support the inclusion of CRMO in the group of autoinflammatory diseases. The higher mRNA levels of *CASP-1* and *pro-IL-1β* found in patients during remission suggest the presence of a sustained proinflammatory state in CRMO patients. The mechanisms leading to this proinflammatory state, as well to activation of the inflammasome in vitro hyper-response are unknown: in particular, possible contribution of genetics have remained unclarified. In addition, we found high expression of IL-6 in bone biopsies from CRMO patients (Figure 2G^I^), suggesting that IL-6 produced at inflammatory sites may be the source of the circulating measurable cytokine in the blood. Circulating IL-6 levels were significantly higher in CRMO patients during active disease. In cryopirinopathies, in which abnormal IL-1 secretion drives the cytokine cascade and all clinical and laboratory features, IL-6 production in tissues and high circulating levels have been shown to be completely IL-1β dependent [10,18].

We have also shown that osteoclasts *in vivo* express inflammasome-related proteins. This is consistent with data in the literature [19-21] and a publically available expression profile database, showing detectable mRNA levels of inflammasome components in osteoclasts differentiated *in vitro*[22]. However, the function of the inflammasome in osteoclasts is not known yet. In this respect, future studies in osteoclasts from patients with Majeed syndrome might represent the ideal model to investigate, in the context of a genetic abnormality, the presence of abnormal function of the osteoclasts.

## Conclusions

We have shown increased IL-1β secretion by peripheral blood mononuclear cells of CRMO patients during active disease and expression of inflammasome components by CRMO and control osteoclasts *in vivo*. These results further support a role for autoinflammation in CRMO.

## Abbreviations

CRMO: Chronic recurrent multifocal osteomyelitis; PBMCs: Peripheral blood mononuclear cells; IL: Interleukin; TLR: Toll-like receptor; LPS: Lipopolysaccharides; ASC: Apoptosis-associated Speck-like protein containing a CARD; CASP-1: Caspase-1; NLRP3: Nod-like receptor protein-3; ATP: Adenosine triphosphate; CRP: C-reactive protein; NSAID: Non steroidal anti-inflammatory drugs; ESR: Erythrocyte sedimentation rate; DIRA: Deficiency of interleukin-1 receptor antagonist; SAPHO: Synovitis-acne-pustulosis-hyperostosis-osteitis syndrome; JIA: Juvenile idiopathic arthritis.

## Competing interests

The authors have declared no conflicts of interest.

## Authors’ contributions

All authors were involved in the design of the study. RS performed stimulation of PBMCs ex vivo, cytokine measurement by ELISA, Real-time PCR analyses and drafted the manuscript, under the supervision and with the collaboration of GP. AI was responsible for the collection of data and provided statistical analysis of the data. RDV and MP were responsible for Immunohistochemical analyses. LBL and AT contributed to the study design and analysis and interpretation of the data. FDB and GP conceived the study, oversaw the project and critically reviewed the manuscript. All the authors have contributed to the writing of the manuscript and have seen and approved the final version of the manuscript.

## References

[B1] Giedion AHW Masel LF Vischer D [Subacute and chronic “symmetrical” osteomyelitis] Ann Radiol (Paris) 1972 15 3 329 342 4403064

[B2] Del Fattore A Teti A The tight relationship between osteoclasts and the immune system Inflamm Allergy Drug Targets 2012 11 181 187 10.2174/187152812800392733 22280239

[B3] Ferguson PJ El-Shanti HI Autoinflammatory bone disorders Curr Opin Rheumatol 2007 19 5 492 498 10.1097/BOR.0b013e32825f5492 17762617

[B4] Ferguson PJ Chen S Tayeh MK Ochoa L Leal SM Pelet A Munnich A Lyonnet S Majeed HA El-Shanti H Homozygous mutations in LPIN2 are responsible for the syndrome of chronic recurrent multifocal osteomyelitis and congenital dyserythropoietic anaemia (Majeed syndrome) J Med Genet 2005 42 7 551 557 10.1136/jmg.2005.030759 15994876 PMC1736104

[B5] Cassel SL Janczy JR Bing X Wilson SP Olivier AK Otero JE Iwakura Y Shayakhmetov DM Bassuk AG Abu-Amer Y Brogden KA Burns TL Sutterwala FS Ferguson PJ Inflammasome-independent IL-1β mediatesutoinflammatory disease in Pstpip2-deficient mice Proc Natl Acad Sci U S A 2014 111 3 1072 1077 10.1073/pnas.1318685111 24395802 PMC3903222

[B6] Hofmann SR Schwarz T Möller JC Morbach H Schnabel A Rösen-Wolff A Girschick HJ Hedrich CM Chronic non-bacterial osteomyelitis is associated with impaired Sp1 signaling, reduced IL10 promoter phosphorylation, and reduced myeloid IL-10 expression Clin Immunol 2011 11 317 327 21925952 10.1016/j.clim.2011.08.012

[B7] Lachmann HJ Quartier P So A Hawkins PN The emerging role of interleukin-1beta in autoinflammatory diseases Arthritis Rheum 2011 63 314 324 10.1002/art.30105 20967858

[B8] Rathinam VA Vanaja SK Fitzgerald KA Regulation of inflammasome signaling Nat Immunol 2012 13 333 342 10.1038/ni.2237 22430786 PMC3523703

[B9] Gattorno M Tassi S Carta S Delfino L Ferlito F Pelagatti MA D’Osualdo A Buoncompagni A Alpigiani MG Alessio M Martini A Rubartelli A Pattern of interleukin-1beta secretion in response to lipopolysaccharide and ATP before and after interleukin-1 blockade in patients with CIAS1 mutations Arthritis Rheum 2007 56 9 3138 3148 10.1002/art.22842 17763411

[B10] Goldbach-Mansky R Dailey NJ Canna SW Gelabert A Jones J Rubin BI Kim HJ Brewer C Zalewski C Wiggs E Hill S Turner ML Karp BI Aksentijevich I Pucino F Penzak SR Haverkamp MH Stein L Adams BS Moore TL Fuhlbrigge RC Shaham B Jarvis JN O’Neil K Vehe RK Beitz LO Gardner G Hannan WP Warren RW Horn W Neonatal-onset multisystem inflammatory disease responsive to interleukin-1beta inhibition N Engl J Med 2006 355 6 581 592 10.1056/NEJMoa055137 16899778 PMC4178954

[B11] Rosengren S Mueller JL Anderson JP Niehaus BL Misaghi A Anderson S Boyle DL Hoffmann HM Monocytes from familial cold autoinflammatory syndrome patients are activated by mild hypothermia J Allergy Clin Immunol 2007 119 4 991 996 10.1016/j.jaci.2006.12.649 17320940 PMC4322003

[B12] Aksentijevich I Masters SL Ferguson PJ Dancey P Frenkel J Van Royen-Kerkhoff A Laxer R Tedgård U Cowen EW Pham TH Booty M Estes JD Sandler NG Plass N Stone DL Turner ML Hill S Butman JA Schneider R Babyn P El-Shanti HI Pope E Barron K Bing X Laurence A Lee CC Chapelle D Clarke GI Ohson K Nicholson M An autoinflammatory disease with deficiency of the interleukin-1-receptor antagonist N Engl J Med 2009 360 23 2426 2437 10.1056/NEJMoa0807865 19494218 PMC2876877

[B13] Lachmann HJ Lowe P Felix SD Rordorf C Leslie K Madhoo S Wittkowski H Bek S Hartmann N Bosset S Hawkins PN Jung T In vivo regulation of interleukin 1beta in patients with cryopyrin-associated periodic syndromes J Exp Med 2009 206 5 1029 1036 10.1084/jem.20082481 19364880 PMC2715040

[B14] Pascual V Allantaz F Arce E Punaro M Banchereau J Role of interleukin-1 (IL-1) in the pathogenesis of systemic onset juvenile idiopathic arthritis and clinical response to IL-1 blockade J Exp Med 2005 201 9 1479 1486 10.1084/jem.20050473 15851489 PMC2213182

[B15] Colina M Pizzirani C Khodeir M Falzoni S Bruschi M Trotta F Di Virgilio F Dysregulation of P2X7 receptor-inflammasome axis in SAPHO syndrome: successful treatment with anakinra Rheumatology (Oxford) 2010 49 7 1416 1418 10.1093/rheumatology/keq074 20299381

[B16] Herlin T Fiirgaard B Bjerre M Kerndrup G Hasle H Bing X Ferguson PJ Efficacy of anti-IL-1 treatment in majeed syndrome Ann Rheum Dis 2013 72 410 413 10.1136/annrheumdis-2012-201818 23087183 PMC3660147

[B17] Eleftheriou D Gerschman T Sebire N Woo P Pilkington CA Brogan PA Biologic therapy in refractory chronic non-bacterial osteomyelitis of childhood Rheumatology (Oxford) 2010 49 1505 1512 10.1093/rheumatology/keq122 20430869

[B18] Hoffman HM Rosengren S Boyle DL Cho JY Nayar J Mueller JL Anderson JP Wanderer AA Firestein GS Prevention of cold-associated acute inflammation in familial cold autoinflammatory syndrome by interleukin-1 receptor antagonist Lancet 2004 364 1779 1785 10.1016/S0140-6736(04)17401-1 15541451 PMC4321997

[B19] Grol MW Panupinthu N Korcok J Sims SM Dixon SJ Expression, signaling, and function of p2x7 receptors in bone Purinergic Signal 2009 5 205 221 10.1007/s11302-009-9139-1 19224395 PMC2686829

[B20] Okahashi N Koide M Jimi E Suda T Nishihara T Caspases (interleukin-1beta-converting enzyme family proteases) are involved in the regulation of the survival of osteoclasts Bone 1998 23 33 41 10.1016/S8756-3282(98)00069-6 9662128

[B21] Yao Z Xing L Qin C Schwarz EM Boyce BF Osteoclast precursor interaction with bone matrix induces osteoclast formation directly by an interleukin-1-mediated autocrine mechanism J Biol Chem 2008 283 9917 9924 10.1074/jbc.M706415200 18250170 PMC2442286

[B22] Vuorikoski Transcription profiling of human osteoclasts, dendritic cells, macrophages and endothelial cells derived from CD14+ peripheral blood mononuclear cells 2006 Available on line: http://www.ebi.ac.uk/microarray-as/ae/browse.html?keywords=E-MEXP-916. In. 2006/11/22 ed

